# Association between mental illness and blood pressure variability: a systematic review

**DOI:** 10.1186/s12938-022-00985-w

**Published:** 2022-03-21

**Authors:** Nur Husna Shahimi, Renly Lim, Sumaiyah Mat, Choon-Hian Goh, Maw Pin Tan, Einly Lim

**Affiliations:** 1 Department of Biomedical Engineering, Faculty of Engineering, University of Malaya, 50603 Kuala Lumpur, Malaysia; 2 Ageing and Age-Associated Disorders Research Group, Faculty of Medicine, University of Malaya, 50603 Kuala Lumpur, Malaysia; 3 UniSA Clinical and Health Sciences, University of South Australia, Adelaide, 5000 Australia; 4 Centre for Healthy Aging and Wellness, Faculty of Health Sciences, Universiti Kebangsaan Malaysia, 50300 Kuala Lumpur, Malaysia; 5 Department of Mechatronics and BioMedical Engineering, Lee Kong Chian Faculty of Engineering and Science, Universiti Tunku Abdul Rahman, Bandar Sungai Long, 43200 Kajang, Selangor Malaysia

**Keywords:** Mental disorder, Blood pressure variability, Autonomic nervous system, Spectral analysis, Time-domain analysis

## Abstract

**Background:**

Mental illness represents a major global burden of disease worldwide. It has been hypothesised that individuals with mental illness have greater blood pressure fluctuations that lead to increased cardiovascular risk and target organ damage. This systematic review aims to (i) investigate the association between mental illness and blood pressure variability (BPV) and (ii) describe methods of BPV measurements and analysis which may affect pattern and degree of variability.

**Methods:**

Four electronic databases were searched from inception until 2020. The quality assessment was performed using STROBE criteria. Studies were included if they investigated BPV (including either frequency or time domain analysis) in individuals with mental illness (particularly anxiety/generalised anxiety disorder, depression/major depressive disorder, panic disorder and hostility) and without hypertension. Two authors independently screened titles, abstracts and full texts. A third author resolved any disagreements.

**Results:**

Twelve studies met the inclusion criteria. Three studies measured short-term BPV, two measured long-term BPV and seven measured ultra-short-term BPV. All studies related to short-term BPV using ambulatory and home blood pressure monitoring found a higher BPV in individuals with depression or panic disorder. The two studies measuring long-term BPV were limited to the older population and found mixed results. Mental illness is significantly associated with an increased BPV in younger and middle-aged adults. All studies of ultra-short-term BPV using standard cardiac autonomic assessment; non-invasive continuous finger blood pressure and heart rate signals found significant association between BPV and mental illness. A mixed result related to degree of tilt during tilt assessment and between controlled and spontaneous breathing were observed in patients with psychological state.

**Conclusions:**

Current review found that people with mental illness is significantly associated with an increased BPV regardless of age. Since mental illness can contribute to the deterioration of autonomic function (HRV, BPV), early therapeutic intervention in mental illness may prevent diseases associated with autonomic dysregulation and reduce the likelihood of negative cardiac outcomes. Therefore, these findings may have important implications for patients' future physical health and well-being, highlighting the need for comprehensive cardiovascular risk reduction.

## Introduction

Mental illness, such as anxiety, depression and bipolar disorder, poses a significant global disease burden [64]. The presence of mental illness is associated with increased morbidity and mortality [36]. Cardiovascular disease is the most common cause of death among individuals with mental illness [15, 36, 50]. This led to the hypothesis that individuals with mental illness have greater blood pressure fluctuations resulting in increased cardiovascular risk and target organ damage [36, 40].

Impaired autonomic function, as reflected in both sympathetic and parasympathetic activity of the autonomic nervous system, is associated with an increased risk of cardiovascular diseases [14]. Conventional methods to assess autonomic function include measurement of heart rate and blood pressure changes in response to a series of challenge manoeuvers. These methods are relatively crude and lack sensitivity, and therefore not routinely used in practice [11]. Newer methods of measuring heart rate and blood pressure changes include measurement of heart rate variability (HRV) and blood pressure variability (BPV). These methods require minimal patient cooperation and have a high level of sensitivity.

HRV is an assessment of beat-to-beat variation in the heart, and is increasingly used because it is simple to measure and is a reliable indicator for autonomic function [4, 40]. Based on studies on heart rate estimation, electrocardiogram (ECG) signals will first undergo a pre-processing step that includes denoising, segmentation, and filtering to remove any undesirable noise or artefacts [49]. The other purpose of filtering is to emphasise the heartbeat peaks (QRS process) of the ECG signals so that the distance between consecutive peaks can be measured (i.e. each R peak corresponds to a heartbeat) [51]. Heart rate and heart rate variability values can be estimated once these steps are completed. A review of heart rate variability and psychopathology suggested that a constantly changing heart rate is a sign of healthy regulatory systems that can successfully adjust to environmental and psychological challenges [47]. Reduced HRV, on the other hand, indicates that the body's stress response is not optimal, potentially exacerbating the negative effects of chronic stress and increasing the risk of stress-related medical conditions. Previous studies reported that HRV indices, as provided by telemedicine and remote healthcare applications [51] were significantly reduced in patients with depression [31, 53] and anxiety disorders, including generalised anxiety disorder (GAD), social anxiety disorder, panic disorder and post-traumatic stress disorder [16, 32]. Thus, we can conclude from a number of studies on HRV and mental illness that lower HRV in patients with mental illness is associated with poorer cardiovascular health outcomes and a variety of vascular diseases [2].

On the other hand, BPV refers to fluctuations in blood pressure that occur within several minutes, over a 24-h period or a longer period of time (several years) [45]. While blood pressure fluctuations over a 24-h period are normally obtained using non-invasive ambulatory blood pressure recorders, continuous, beat-to-beat blood pressure measurements are acquired using the photoplethysmographic (PPG) technique. Long-term BPV has been associated with stroke and coronary events in high-risks patients [55], while visit-to-visit short-term BPV is a prognostic indicator for cardiovascular mortality in patients with hypertension [44]. Although the association between BPV and coronary diseases has been widely reported, the mechanisms linking these two are unclear due to the dynamic nature of blood pressure, which fluctuates with environmental stimulations and daily life challenges [44, 46]. According to Parati et al. [46], some modifiable risk factors that may alter BPV and affect cardiovascular health outcomes include subjects’ reactivity to emotional stimuli (.e.g., mental state, physiological stress) and behavioural factors (e.g., level of physical activity, sleep cycles, postural changes).

While BPV has been widely studied in hypertension [34, 62], less is known about its relationship with mental illness. Several studies have reported an increase in BPV in individuals with mental illness, which has been linked to an increase in their cardiovascular risk and target organ damage. However, conflicting findings with regard to BPV in patients with psychological disorders have been reported. This may be caused by inconsistencies in the study design among different clinical studies, measurement techniques and experimental procedures as well as types of BPV analysis. Therefore, the current systematic review aims to (i) investigate the association between mental illness and BPV; and (ii) provide an in-depth analysis on the study design, blood pressure (BP) measurement techniques and assessment intervals as well as types of BPV indices used in each study. This would help improve the design of future clinical trials, which aim to identify the relationship between BPV and psychological disorders and use BPV as early indicators of cardiovascular diseases in these patients.

## Results

### Description of studies

**Fig. 1:**
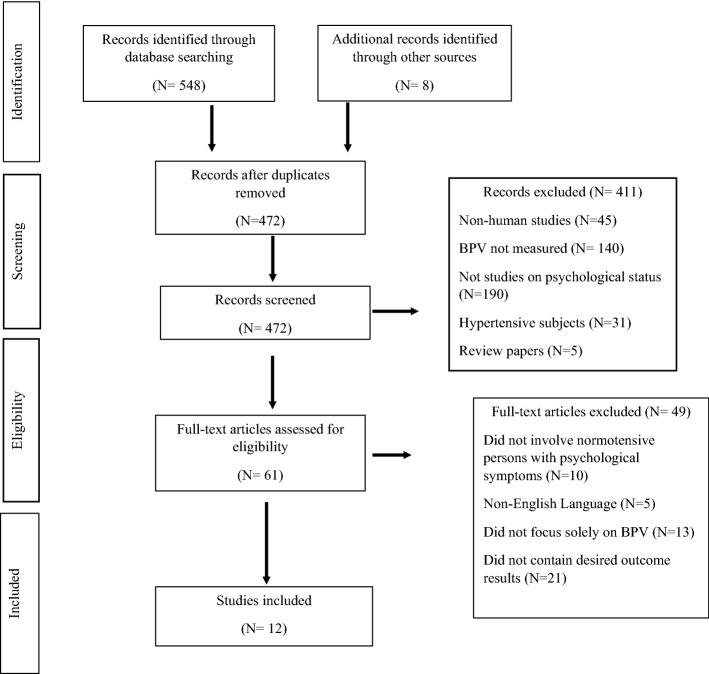
Flowchart of the study identification and selection process. *BPV* blood pressure variability

Figure 1 shows the study identification and selection process. Of the 556 records identified through database searching and through other sources, 472 studies were potentially eligible after removal of duplicates. Following screening of title, abstract and full-text articles, 12 articles fulfilled the inclusion criteria.

### Risk of bias

Table 1 summarises the quality assessment of all studies included in this review (*n* = 12). The majority of the studies provided detailed information about the research framework. Eleven studies provided a clear description of background of study, aims, objectives and hypotheses in the introduction. No studies reported the sample size calculation, and only two studies addressed missing data [57, 65].

**Table 1:** The assessment quality for potential risk of bias

Author (year)	Checklist questions																Score
	**1**	**2**	**3**	**4**	**5**	**6**	**7**	**8**	**9**	**10**	**11**	**12**	**13**	**14**	**15**	**16**	
Bystritsky & Shapiro, (1992)	**/**	**/**	**/**	**/**	**/**	**/**		**/**		**/**		**/**	**/**	**/**	**/**	**/**	13/16
Piccirillo et al*.*, (1997)	**/**	**/**	**/**	**/**	**/**	**/**		**/**		**/**	**/**	**/**	**/**	**/**	**/**		13/16
Carels et al., (2000)	**/**	**/**	**/**	**/**	**/**	**/**		**/**		**/**		**/**	**/**	**/**	**/**	**/**	13/16
Virtanen et al*.*, (2003)	**/**	**/**	**/**	**/**	**/**	**/**		**/**		**/**	**/**	**/**	**/**	**/**	**/**	**/**	14/16
Otsuka et al*.*, (2004)	**/**	**/**	**/**	**/**	**/**			**/**		**/**			**/**	**/**	**/**		10/16
Yeragani et al*.*, 2004)	**/**	**/**	**/**	**/**	**/**	**/**		**/**	**/**	**/**			**/**	**/**	**/**		12/16
Davydov et al*.*, (2007)	**/**	**/**	**/**	**/**	**/**	**/**		**/**		**/**	**/**	**/**	**/**	**/**	**/**	**/**	14/16
Martinez et al*.*, (2010)	**/**	**/**	**/**	**/**	**/**	**/**		**/**		**/**		**/**	**/**	**/**	**/**		12/16
Vasudev et al., (2011)	**/**	**/**	**/**	**/**	**/**	**/**		**/**		**/**		**/**	**/**	**/**	**/**	**/**	13/16
Alici et al*.*, (2014)	**/**			**/**	**/**			**/**		**/**		**/**	**/**			**/**	8/16
Phillip J. Tully & Tzourio, (2017)	**/**	**/**	**/**	**/**	**/**	**/**		**/**	**/**	**/**		**/**	**/**	**/**	**/**	**/**	14/16
Philip J. Tully, Debette, & Tzourio, (2017)	**/**	**/**	**/**	**/**	**/**	**/**		**/**		**/**		**/**	**/**	**/**	**/**	**/**	13/16
Number of fulfilling each item	12	11	11	12	12	10	0	12	2	12	3	10	12	11	11	8	
Percentage (%)	100	91.7	91.7	100	100	83.3	0	100	16.7	100	25	83.3	100	91.7	91.7	66.7	

**Quality Assessments Questions**

**Introduction**

1.Does the abstract provide an informative and balanced summary of what was done and what was found?

2.Are the primary outcomes to be measured clearly stated in the Introduction section?

3.Is the study's hypothesis and objectives clearly stated?

**Methodology**

4.Does the study clearly describe the methodology/protocol of studies which includes the setting, locations, periods of recruitment and data collection?

5.Does the characteristics of participants included in the study clearly described?

6.Does the distributions of outcomes, exposures, predictors, potential confounders, and effect modifiers in each group of subjects to be compared clearly described?

7.Is the calculation of study size/sample size reported?

8.Were the statistical tests used to access the main outcomes appropriate?

9.Does any missing data were addressed in the study?

**Results**

10.Does the study reported the number of individuals included in the study?

11.Does the study clearly indicate the characteristics of study participants (demographic) including the number of missing data for each variable of interest?

12.Are the main findings of the study clearly described?

**Discussion**

13.Does the study summarise the key results with reference to study objectives?

14.Does the study discussed the limitations of the study, taking into account sources of potential bias?

15.Does the study interpreted overall results considering objectives, limitations, multiplicity of analyses and results from similar studies/relevant evidence?

**Other Information**

16.Does the study state the source of funding or the role of funders for the present study?

### Population characteristics

Table 2 summarises the characteristics of the 12 studies. Five studies involved older adults aged 55 years and above [41, 48, 56, 57, 59], whilst the remaining seven studies assessed BPV in the younger to middle-aged population aged 18 to 46 years [1, 6, 7, 13, 35, 61, 65]. All studies included both males and females [6, 13, 41, 56, 57, 59, 65]. The presence and severity of mental health symptoms or the diagnoses of mental illness was assessed using a number of validated assessments or tools. The assessment tools and the methods of diagnoses are listed in Table 2.

**Table 2:** Summary of studies for psychological status or mental illness on types of BPV

Author citation	Psychological symptoms or conditions	Participants with psychological status	Healthy control subjects	Psychiatric assessment/tools	BPV measures	Setting and time of the assessment	Findings
Short Term: Time Domain Analysis							
Carels et al., (2000)	Depression or MDD	162 (Age: 25–45 years)	-	**1.**Beck Depression Inventory (BDI) **2.**Trait Anxiety Inventory (TAI) **3.**Social Support Questionnaire-Short Form (SSQ6) **4.**Daily Stress Inventory (DSI)	Mean BP Standard deviation (SD)	Setting: Clinic Time: - Setting: Home Time: Workday period (four times per hour)	- ↑ (SBP, DBP) in High Emotional Responsivity group - NS (SBP, DBP) in Low Emotional Responsivity group
Otsuka et al*.*, (2004)	Depression or MDD	72 Aged: between 24 and 79 years (mean age: 56.8 ± 11.3 years)	120 Aged: between 24 and 79 years (mean age: 56.8 ± 11.3 years)	**1.**Depression Rating Scale	mean ± SD SBP DBP Pulse Pressure (PP) Double Product (DP)	Setting: Home Time: 7 day/ 24-h	-↑ (24-h average SBP, DBP) in depressed subjects as compared to controls - ↓ (DBP dipping), - NS (SBP dipping ratio)
Alici et al*.*, (2014)	PD	25 Age: 31.6 ± 12.2 years	25 Age: 34 ± 11 years	**1.**DMS-IV (Diagnostic and Statistical Manual of Mental Disorders, Fourth Edition)	Average SBP Average DBP mBP (mean BP)	Setting: Home Time: a) Diurnal reading: 0800–2200 h (activity that take place in the daytime) b) Nocturnal reading: 2200–0800 h (activity that take place at nighttime)	- NS (24-h SBP) between PD and control -↑ (24-h DBP) in PD -↓ (reduction in nighttime SBP, DBP, mBP) in PD
Long Term: Time Domain Analysis							
Phillip J. Tully & Tzourio, (2017)	Depression or MDD	1454 elderly (59% Female) Age: 78.5 ± 3.78 years	-	**1.**MINI International Neuropsychiatric Interview (structured clinical interview)	CV SD mean SBP SBPV DBPV	Setting: Home Time: Morning (< 1 h after awaking and before taking any drugs); evening (measures must be close to bedtime) Setting: Clinic Time: Prior to HBPM, and again at year 2, 5, and 8 (no specific time mention)	-NS (8 years SBPV, DBPV) in MDD
Philip J. Tully, Debette, & Tzourio, (2017)	Depression or MDD	2812 elderly Age: ≥ 65 years LOD symptomatic: 105 LOD asymptomatic: 200 EOD symptomatic: 51 EOD asymptomatic: 74	No depression disorder, Symptomatic: 190 Asymptomatic: 2192	**1.**MINI International Neuropsychiatric Interview (structured clinical interview) **2.**Centre for Epidemiological Studies-Depression (CESD) scale **3.**DMS-IV (Diagnostic and Statistical Manual of Mental Disorders, Fourth Edition) **4.**Mini Mental State Examination (MMSE)	Coefficient of variation (CV) Mean BPV Mean SBP	Setting: Clinic Time: Three measure were taken at each clinic visit in the first 4 years (up to maximum of 9 BP readings from baseline, year 2, and year 4)	- ↑ (SBPV) in LOD after adjustment for covariates -↑(SBPV) in EOD symptomatic & LOD asymptomatic in analyses adjusted for age, sex, education - NS (DBPV) depression group
Phillip J. Tully & Tzourio, (2017)	Anxiety or Generalised Anxiety Disorder (GAD)	1454 elderly (59% Female) Age: 78.5 ± 3.78 years	-	**1.**MINI International Neuropsychiatric Interview (structured clinical interview)	CV SD mean SBP SBPV DBPV	Setting: Home Time: Morning (< 1 h after awaking and before taking any drugs); evening (measures must be close to bedtime) Setting: Clinic Time: Prior to HBPM, and again at year 2, 5, and 8 (no specific time mention)	- ↑ (8 years SBPV) in GAD - NS (8 years DBPV) in GAD
Ultra-short Term: Time Domain Analysis							
Davydov et al*.*, (2007)	Depression or MDD	28 Age: 20–52 years	28	**1.**MINI International Neuropsychiatric Interview (structured clinical interview) **2.**Hamilton Depression Scale (HAM-D17) **3.**Cook–Medley Hostility Inventory (CM) **4.**Spielberger Anger Expression Scale **5.**Spielberger Trait Anxiety Inventory (STAI) **6.**Marlowe–Crowne Scale of Social Desirability (MC)	Mean SBP ± MSDp (mean of successive difference)	Setting: Soundproof laboratory Time: -	- ↑ (mSBP, MSD-SBP) - ↑ (SBP set point for up and down baroreflex sequence)
Vasudev et al., (2011)	Depression or MDD	41 Age: > 60 years	32 Age: > 60 years	**1.** Montgomery–Asberg Depression rating scale (MADRS) **2.** Hospital Anxiety and Depression Scale (HADS) **3.** Mini Mental State Examination (MMSE) **4.** Cumulative Illness Rating Scale, geriatrics (CIRS-G)	Mean Standard deviation (SD)	-	- ↓(SBP) in depressed group - No difference in DBP reduction - No differences in SBP drop between late-onset depression (LOD) and early onset depression (EOD)
Bystritsky & Shapiro, (1992)	PD	6 Age: 18–46 years	6 Age: 18–52 years	**1.**Diagnostic and Statistical Manual of Mental Disorders-III R criteria **2.**4-D Anxiety Scale **3.**Acute Panic Inventory (API) **4.**Hamilton Anxiety Scale (HAM-A) **5.**Hamilton Depression Scale (HAM-D) **6.**Subjective Anxiety Rating (SAR) **7.**Visual Analog Scale of Anxiety	Mean, Standard deviation (SD)	Setting: Laboratory (sound-attenuated chamber) Time: two 1-h session (2^nd^ session was conducted 3 days after the initial meetings	-↓ (SBP) in PD - ↑ (DBP) in PD - ↑ (SBP, DBP degree of fluctuation) in PD - ↑ (SD SBP variability) in PD - ↑ (breathing irregularities) in PD patients responded to CO_2_ inhalation
Yeragani et al*.*, (2004)	PD	21 Age: 35 ± 6 years	18 Age: 33 ± 7 years	**1.**Diagnostic and Statistical Manual of Mental Disorders-III R criteria **2.**Spielberger’s State Anxiety Inventory (SAI)	**Non-Linear Measure** LLE-SBP LLE-DBP LLE-HR	**-**	. **Non-Linear Measure** - ↑ (supine LLE-SBP, LLE-DBP) in PD - ↑ (Supine & standing LLE-SBP/LLE-HR) in PD - ↑ (Supine & standing LLE-DBP/LLE-HR) in PD
Piccirillo et al*.*, (1997)	Anxiety or GAD	68 **Subgroup:** 36 (one anxiety symptom) Age: 56.8 ± 3.6 years 32 (two or more anxiety symptoms) Age: 55.0 ± 2.9 years	49 Age: 55.8 ± 2.8 years	**1.**Anxiety Symptom Scale (translated into Italian)	mean-SBP mean-DBP	Setting: Quite and comfortable environment (24^0^C) Time: 8.30 am	- Similar resting/baseline mean SBP for all groups - ↑ (mSBP) in two or more anxiety symptoms during tilt period - NS (mSBP) in control during tilt period - ↑ (baseline mDBP) in subjects with two or more anxiety symptoms - ↑ (mean DBP) in all groups during tilt, but subjects with two or more anxiety symptoms had a ↓ percentage increase
Ultra-short Term: Frequency Domain Analysis							
Yeragani et al*.*, (2004)	PD	21 Age: 35 ± 6 years	18 Age: 33 ± 7 years	**1.**Diagnostic and Statistical Manual of Mental Disorders-III R criteria **2.**Spielberger’s State Anxiety Inventory (SAI)	**Linear Measure** Mean BP TP VLF LF HF BP*vi*	**-**	**Linear Measure** -↑ (Standing BP*vi* SBP, DBP) in PD groups -↓ (TP, LF of SBP and DBP) in PD group during controlled breathing at 20 bpm condition
Martinez et al*.*, (2010)	PD	30 Age: 32.5 ± 8.9 years	10 Age: 27.8 ± 7.4 years	**1.**DMS-IV (Diagnostic and Statistical Manual of Mental Disorders, Fourth Edition) **2.**CGI-S for severity of illness (Clinical Global Impression Scale) **3.**HAM-A (Hamilton Rating Scale for Anxiety) **4.**Hamilton Rating Scale for Depression **5.** Panic Disorder Severity Scale (PDSS) **6.** Acute Panic Inventory (API) **7.**10-point Anxiety Scale Score	LF-SBP LF-DBP HF-SBP HF-DBP LF/HF TP	**-**	- ↓ (HF-SBP, TP-SBP) in PD during controlled breathing - NS (LF-SBP, LF-DBP, HF-DBP, LF/HF) in PD patients
Piccirillo et al*.*, (1997)	Anxiety or GAD	68 **Subgroup:** 36 (one anxiety symptom) Age: 56.8 ± 3.6 years 32 (two or more anxiety symptoms) Age: 55.0 ± 2.9 years	49 Age: 55.8 ± 2.8 years	**1.**Anxiety Symptom Scale (translated into Italian)	LF/HF LF-SBPV VLF-DBPV HF-DBPV TP-SBPV	Setting: Quiet and comfortable environment (24^0^C) Time: 8.30 am	- ↓ (LF-SBPV) at rest in control - ↑ (VLF, TP) with two or more anxiety symptoms after tilt - ↓ (LF) in subjects with one anxiety symptoms after tilt - ↑ (VLF DBPV, HF DBPV) in control at rest
Virtanen et al*.*, (2003)	Anxiety or GAD	150 Age: 35–64 years	-	**1.**Brief Symptom Inventory 37-item (BSI-37) **2.**Spielberger State-Trait Anger Expression Inventory (STAXI) **3.**Toronto Alexithymia Scale (TAS-26)	LF-BPV HF-BPV LF power of SAP variability LF power of DAP variability HF power of SAP variability HF power of DAP variability	Setting: In an isolated examination room at a stable temperature between 20^0^ and 22^0^C Time: 8.30am- 12 pm	- ↑ (LF-BPV) in anxiety groups - ↑ (anxiety), ↑ (LF- systolic arterial pressure variability - NS (HF-diastolic arterial pressure variability) in anxiety
Virtanen et al*.*, (2003)	Hostility	150 Age: 35–64 years	-	**1.**Brief Symptom Inventory 37-item (BSI-37) **2.**Spielberger State-Trait Anger Expression Inventory (STAXI) **3.**Toronto Alexithymia Scale (TAS-26)	LF-BPV HF-BPV LF power of SAP variability LF power of DAP variability HF power of SAP variability HF power of DAP variability	Setting: In an isolated examination room at a stable temperature between 20^0^ and 22^0^C Time: 8.30am- 12 pm	- ↑ (LF-BPV) in hostility group - ↑ (hostility), ↑ (LF-diastolic arterial pressure variability - NS (HF-diastolic arterial pressure variability) in hostility group

Key: PD, panic disorder; MDD, major depressive disorder; GAD, general anxiety disorder; HBPM, home blood pressure monitoring; LOD, late-onset depression; EOD, early onset depression; SD, standard deviation; CV, coefficient variation; BP, blood pressure; SBP, systolic blood pressure; DBP, diastolic blood pressure; BPV, blood pressure variability; SBPV, systolic blood pressure variability; DBPV, diastolic blood pressure variability; LLE-SBP, largest Lyapunov exponent-systolic blood pressure; LLE-DBP, largest Lyapunov exponent-diastolic blood pressure; LLE-HR, largest Lyapunov exponent-heart rate; LF, low frequency; HF, high frequency; VLF, very low frequency; TP, total power; LF/HF, ratio of InLF and InHF; BPvi, Log_10_ ((BP detrended variance/mean BP^2^)/(HR detrended variance/mean HR^2^)); RRI, RR-interval; SAP, systolic arterial pressure; DAP, diastolic arterial pressure

### Psychiatric assessment validation

Of the 12 included studies, three aimed at gaining additional insights into the psychological state using the short-term BPV analysis method [1, 7, 41]. Alici et al. [1] diagnosed patients with panic disorder using the DSM-IV criteria (fourth edition of the Diagnostic and Statistical Manual of Mental Disorders), while the study by Carels et al. [7] used the Beck Depression Inventory (BDI), Trait Anxiety Inventory (TAI), Social Support Questionnaire-Short Form (SSQ6) and Daily Stress Inventory (DSI) assessments to distinguish between emotionally responsive and unresponsive individuals. The third study [41] identified subjects with depression using the 15-item Depression rating scale (through a self-administered questionnaire).

Both long-term BPV studies identified patients with late-onset depression [56], generalised anxiety disorder and depression disorder [57] using the MINI International Neuropsychiatric Interview (MINI) screening tool. The interviews were conducted face-to-face by trained clinical psychologists [56].

Of the seven ultra-short-term BPV studies, four involved psychiatrists to diagnose psychological disorders, and trained clinical interviewers to administer structured clinical interviews with DSM-III-R (Diagnostic and Statistical Manual of Mental Disorders, third addition, revised) [6, 35, 65] and DSM-IV. Two studies evaluated the severity of anxiety states using the Spielberger’s State Anxiety Inventory (SAI) [65] and Anxiety Symptom’s scale [48]. Another study [13] performed face-to-face structured clinical interviews based on the MINI International Neuropsychiatric Interview screening tool to identify patients with major depressive disorder. Lastly, self-reported questionnaires, which include Brief Symptoms Inventory (BSI), Spielberger State–Trait Anger Expression Inventory (STAXI) and Toronto Alexithymia scale (TAS-26), were administered by trained researchers in the study by Virtanen et al. [61].

### Autonomic nervous system (ANS) testing

A number of physiological assessments were used to determine the HRV and BPV. Studies which measured short-term BPV [1, 7, 41] and long-term BPV [56, 57] utilised either Ambulatory Blood Pressure Monitoring (ABPM) or Home Blood Pressure Monitoring (HBPM). Studies which measured ultra-short-term BPV (second/minute) or beat-to-beat variation used the tilt table or orthostatic challenge test to capture continuous blood pressure (BP) and heart rate (HR) recordings for participants [6, 13, 35, 48, 59, 61, 65]. Out of seven studies on ultra-short-term BPV, we identified two studies that used passive tilt table with a known tilt angles of 60° [35] and 90^o^ [48] for 15-min periods. Two ultra-short-term studies used active standing [65] for 3-min periods [59], whilst the remaining studies used continuous HR and BP measurements from either supine or sitting conditions [6, 13, 61]. Four studies using ultra-short-term also included controlled breathing at 15 breaths/minutes [35, 61], nine breaths/minutes [48] and 12 and 20 breaths/minutes [65].

### Blood pressure variability and indices used

Three studies measured short-term BPV [1, 7, 41], two studies measured long-term BPV [56, 57] and seven studies measured ultra-short-term BPV or beat-to-beat variation [6, 13, 35, 48, 59, 61, 65]. Three studies which measured short-term BPV [1, 7, 41] and two studies on long-term BPV [56, 57] assessed time domain variability. Of the seven studies that measured ultra-short BPV, two utilised both time domain and frequency domain methods [48, 65], two evaluated the frequency domain [35, 61] and three determined variability in the time domain [6, 13, 59].

Two types of linear analysis on BPV were used: the time domain and frequency domain analysis. For short-term BPV studies, blood pressure and heart rate data were obtained using non-invasive ambulatory blood pressure monitors/recorders (ABPM) over a 24-h period at regular intervals to observe diurnal and nocturnal blood pressure fluctuations in the patients [1, 7, 41]. For example, Alici et al. [1] and Otsuka et al. [41] observed heart rate and blood pressure readings at 20-min interval and 30-min interval, respectively, during the day and at 30-min interval and 60-min interval, respectively, at night. In contrast, both long-term BPV studies [56, 57] acquired the blood pressure readings using the validated digital electronic tensiometer (OMRON). Since all the heart rate and blood pressure data for short- and long-term BPV were recorded using a device, the stored data were downloaded and processed using statistical analysis method (i.e. SPSS) which resulted in time-domain indices such as standard deviation (SD) [21, 22] and coefficient of variation (CV) [33, 57].

Ultrashort-term BPV studies used standard cardiac autonomic assessment (tilt table/active stand), with beat-to-beat blood pressure signals acquired using the photoplethysmographic (PPG) technique, while R–R intervals were acquired based on the electrocardiogram (ECG) signals [35, 48, 61, 65]. The ECG and finger blood pressure waveforms were then pre-processed using custom written software (i.e. PV-WAVE programming language) [35], where filtering, tracing, and denoising were performed to remove any unwanted artefacts [48]. The QRS peaks of the ECG as well as the peaks/troughs of the cyclical blood pressure waveform within every cardiac cycle were then detected using standard derivative or threshold algorithms for the purpose of estimating heart rate and systolic or diastolic blood pressure [48, 65]. Linear detrending technique was also performed before computing the frequency domain indices using the spectral power analysis method [65]. Autoregressive [35, 48, 59] or fast Fourier transform (FFT) algorithms and triangular smoothing [61] were applied on the extracted beat-to-beat heart rate and systolic/diastolic blood pressure data to obtain the frequency-domain BPV indices: very low-frequency (0.04–0.07 Hz), low-frequency (0.07–0.14 Hz), high-frequency (0.14–0.35 Hz), total power (TP) and low-frequency to high-frequency ratio (LF/HF) [19, 22, 48, 65]:

1 $${\text{Total power }}\left( {{\text{TP}}} \right) \, = {\text{ VLF power }} + {\text{ LF power }} + {\text{ HF power,}}$$

2 $$\frac{\mathrm{LF}}{\mathrm{HF}}\mathrm{ratio }=\frac{\mathrm{LF power}}{\mathrm{HF power}},$$

where very low frequency (VLF), low frequency (LF) and high frequency (HF) power were calculated in absolute values.

## Association between mental illness and BPV

### Short term: time domain analysis

Three studies on short-term BPV aimed at gaining additional insight into autonomic function by using time domain analysis [1, 7, 41]. Two studies found a higher 24-h [7] and 7-day/24-h [41] average systolic blood pressure (SBP) and diastolic blood pressure (DBP) among individuals with depressive symptoms. The third study [1] similarly reported a higher 24-h average BP specifically in DBP in patients with panic disorder. Apart from 24-h BP fluctuations, short-term BPV was also observed in terms of nocturnal BP dipping and morning BP surge. Panic disorder was significantly associated with lower reduction in both systolic BP and diastolic BP [1].

### Long term: time domain analysis

Studies on the association between mental illness and BPV using long-term: time domain analysis were limited to the older population. One study [57] found mixed results while another study [56] found significant association between anxiety and BPV.

### Ultra-short term: time domain analysis

Out of the seven studies conducted on ultra-short-term BPV, five presented their findings using parameters within the time domain [6, 13, 48, 59, 65]. Two of the six time domain studies assessed BPV in participants with panic disorder; both studies found increased BPV in patients with panic disorder [6, 65]. Two studies demonstrated increased BPV in individuals with depressive symptoms or major depressive disorder (MDD) [13, 59]. One study found significant association between BPV and anxiety or generalised anxiety disorder [48].

### Ultra-short-term: frequency domain analysis

Of the seven studies which evaluated ultrashort-term BPV, four studies found significant association between BPV and mental illness using frequency domain power spectral analysis [35, 48, 61, 65]. Studies by Martinez et al. [35] and Yeragani et al. [65] highlighted a tilt table test in panic disorder participants. Most studies evaluated ultra-short-term BPV during normal, spontaneous breathing. Additional measurements recorded during controlled breathing were conducted in both studies [35, 65] on panic disorder with controlled breathing leading to differences in BPV. Martinez et al. [35] found that controlled breathing phase (15 breaths/minutes) produced significantly lower high frequency and lower total power of SBP in comparison to spontaneous breathing. Yeragani et al. [65] reported controlled in systolic BPV and diastolic BPV, while Martinez et al. [35] did not report diastolic BPV. Yeragani et al. [65] found significantly lower total and low-frequency power for both systolic BPV and diastolic BPV during controlled breathing phase (20 breaths/minutes) compared to spontaneous breathing.

Two studies found significant association between BPV and anxiety or generalised anxiety disorder in spectral analysis parameters [48, 61]. Piccirillo et al. [48] found mixed results with increased very low frequency and total power spectral density if two or more anxiety symptoms were present after tilting. Another study found that higher scores for anxiety were associated with higher low-frequency systolic BPV, while high-frequency diastolic BPV showed no significance at all in anxiety groups [61]. One ultrashort-term BPV study assessed hostility [61] with increased hostility associated with increased low-frequency diastolic BPV (*p* = 0.001) and increased high-frequency systolic BPV (*p* = 0.033).

## Discussion

Studies using short-term and ultrashort-term BPV supported the hypothesis that young and middle-aged participants with mental illness have dysregulated autonomic function, as reflected by an increased BPV. The association between mental illness and long-term BPV in the older population is less clear.

Short-term BPV which includes day and night time measurements [5] as well as nocturnal BP dipping and morning BP surge [9] indicates blood pressure fluctuations which occur over a 24-h time period. Panic disorder is associated with reduced nocturnal dipping [1]. A reduction in diastolic BP dipping but not systolic BP dipping was observed in individuals with depressive symptoms identified with the depression rating scale [41]. The non-dipper (or reduced nocturnal dipping) patterns observed with panic and depressive symptoms may be a marker of reduced arterial elasticity. Traumatic events, stress or hyperarousal symptoms and poor sleep quality have been associated with changes in nocturnal BP [58]. Stress hormones released by the adrenal glands tend to increase upon sleep onset, resulting in hyperarousal of the sympathetic response at night. Hyperarousal of the sympathetic response results in sleep difficulty, which is commonly associated with psychological illnesses [17, 27, 63]. Reduced baroreflex sensitivity may also explain the presence of non-dipper patterns [24].

Long-term BPV is usually obtained through repeated procedures of BP measurements over days, weeks, months, seasons and even years [45, 52]. Previous studies have shown that generalised anxiety disorder and late-onset depression are associated with higher systolic BPV determined over 8–10 years [56, 57], but major depression is not associated with systolic BPV [56]. The prognostic significance of long-term BPV over short-term BPV remains unclear with factors such as BP treatment and changes in methods of measurement over time likely to confound the overall findings [26, 56].

Ultrashort-term BPV is frequently reflected by the contribution of different humoral systems such as the cardiovascular control system, myogenic response, renin–angiotensin system and endothelium-derived nitric oxide in blood pressure regulation [26]. Previous studies have shown that time domain BPV is significantly associated with panic disorder [65] and anxiety [48]. Higher resting BP has previously been documented in these two groups in studies involving solitary measurement and this observation has been attributed to sympathetic activation and deactivation of vagal activity [60]. Head-up tilting leads to increased sympathetic activation stimulated by the postural challenge, which is usually only demonstrable through frequency domain evaluations manifesting as both increased low-frequency power spectral density and increased low- to high-frequency power density ratio. The percentage increase in these two measurements is reduced in individuals with panic disorder and anxiety symptoms [35, 48, 65]. This may signify reduced sympathetic response due to increased baseline sympathetic activation.

There were significant heterogeneity in both study protocols and sample selections of all studies. The small sample sizes and different time frame may have contributed to the inconsistency in the findings between studies included in this review. The majority of studies included young, middle-aged and older populations; many excluded older adults. The different age groups may also account for the differences in findings between studies. Significant difference in autonomic responses has been observed in older groups compared to younger age groups [43]. Fiske et al. [18] found that increased age is associated with a reduction in neurotransmitter release, which indirectly reduces their mood regulation and the ability to adapt to environmental changes or intrinsic visceral stimuli. Hence, reduction in mood regulation will lead to diminished autonomic reactivity, specifically blood pressure and cerebral blood flow regulation [25].

There is currently a lack of standardisation in terms of protocol for BPV assessment. For instance, there were differences in the tilt angle across the ultrashort-term studies [35, 48]. The duration of the supine position among ultrashort-term studies also differed. Three studies [35, 48, 59] demonstrated a longer supine resting period with at least 10- to 15-min recording, while one study [61] only measured five minutes of supine resting recording. Duration of the baseline recording could play an important role in determining the association between BPV and psychological symptoms. Fluctuations in HR and BP for hostility, for example, could only be detected after a time period long enough to create the interactions between the subjects and their surroundings [54]. Additionally, the relationship between the duration of time for posture recording and autonomic system is still not clear because there is no “gold standard” for tilt-table testing. The different breathing rates employed during postural challenge is likely to result in different in BPV measurements. Controlled breathing may be anxiogenic particularly in individuals with psychological disorders while higher breathing may also lead to hypocapnia resulting from hyperventilation. Therefore, it may not be considered physiological, and hence counterproductive in terms of standardisation of breathing rates to facilitate computational accuracy and reduce noise [23, 35]. Further advancements in both statistical methods and engineering innovations could perhaps resolve this conundrum by measuring breathing rates alongside BP measurements and calculating variability adjusted for spontaneous variabilities in breathing rates which occur within and between individuals.

Our review had some limitations. Firstly, although our review demonstrated changes in BPV in individuals with mental illness, we only included individuals with depression, anxiety, stress and panic disorder. Future studies involving other types of mental illnesses with varying severity, which may affect autonomic function differently, should be considered. Secondly, we did not extract information on medicines use; use of medicines may alter BPV.

## Conclusion

Mental illness is significantly associated with an increased BPV in younger and middle-aged adults. The association between mental illness and BPV is less clear in the older population. Larger studies involving older adults are needed to examine the association between mental illness and BPV.

## Methods

This systematic review is reported according to the Preferred Reporting Items for Systematic Reviews and Meta-analyses 2020 (PRISMA-S) statement [42].

### Search strategy and terminology

Four electronic databases, National Library of Medicine (PubMed), PubMed Central® (PMC), MEDLINE® and CINAHL, were searched from inception until 2020. We identified relevant articles reporting human studies published in English for relevant combinations of the following terms and words in titles and abstracts: “Blood pressure variability”, OR “BPV”, OR “blood pressure changes”, AND “psychological disorders”, OR “stress”, OR “depression”, OR “anxiety”, OR “panic disorder”, OR “worry”. Titles, abstracts and full-text articles were assessed for eligibility using methods recommended in the Joanna Briggs Institute (JBI) systematic review [3]. Titles and abstracts of identified articles were independently screened by two authors (NHS and EL) and any disagreements resolved by a third author (SM). Two authors (NHS and EL) assessed the full-text articles of the potentially eligible studies and a third author (SM) resolved any disagreements. Additional articles were identified by checking the reference lists of full-text articles included in this systematic review.

### Inclusion and exclusion criteria

Inclusion criteria were: (1) studies that investigated BPV in individuals with mental illness; (2) BPV measures included frequency or time domain analysis; and (3) study participants did not have hypertension. We excluded studies that did not meet with the purpose of the review, such as where BPV was not the main focus of the study.

### Data extraction

All papers identified from the initial electronic search process were imported into a reference management software (EndNote Version X9, Clarivate Analytics) where duplicates were removed. Data were extracted by one author (NHS) and crossed checked by a second author (EL) using an electronic data extraction form.

### Outcomes

BPV measurements can be divided into short-term, long-term and ultra-short term. Short-term BPV is defined as blood pressure changes or fluctuations that occur over a 24-h time period (minute-to-minute, hour-to-hour, and day-to-night changes) [5, 44]. Long-term BPV is usually obtained through prolonged periods of measurements over days, weeks, months, seasons and even years [45, 52]. Ultrashort-term BPV is defined as beat-to-beat blood pressure measurement over seconds to minutes [26, 44, 45].

BPV analysis can be separated into time domain and frequency domain analysis. Time-domain analysis measures dispersion of blood pressure values over a given time window, while frequency domain analysis measures blood pressure fluctuations as a function of frequency. Time-domain indices are divided into two categories: (1) simply measures of dispersion of average values over a given time window (e.g., standard deviation and coefficient of variation over 10 min of supine rest); (2) estimation that take accounts the sequence of measurements over time (e.g., average real variability, root mean square of real variability, and standard deviation of real variability, all of these take accounts the beat-to-beat changes). Various time domain indices have been proposed, such as the standard deviation (SD) and coefficient variation (CV).

Frequency-domain indices are obtained through spectral analysis techniques, in which we do not express beat-to-beat BP values as a function of time, but as a function of frequency. It concentrates on revealing the cyclical nature hidden in the series of changing beat-to-beat BP values. The frequency and magnitude of these oscillations are measured, which allows the calculation of the power density for separate frequency ranges. Frequency-domain indices are divided into three main components: very-low frequency (0.016–0.04 Hz), low frequency (0.07–0.14 Hz) and high frequency (0.14–0.35 Hz) [21]. Very low frequency metric is related to the renin–angiotensin system, high-frequency component measures the parasympathetic activity, while low frequency measures the sympathetic activity [35, 48]. The ratio of low frequency and high frequency reflects the sympathovagal balance between the sympathetic and the parasympathetic activities [35].

### Quality assessment

Risk of bias was assessed using the Strengthening the Reporting of Observational Studies in Epidemiology (STROBE) criteria [8]. Only 16 items identifying potential sources of bias relevant to the scope and objectives of our review were selected for reporting. The checklist comprised 5 domains: introduction (item 1–3), research methodology (item 4–9), results (item 10–12), discussion (item 13–15) and other information (item 16). Discrepancies were resolved through discussions between two reviewers (NHS, EL), and there were no disagreements between both reviewers regarding the risk of bias assessment.

## Data Availability

All relevant data are contained within the article.

## Abbreviations

ANS: Autonomic nervous system
BPV: Blood pressure variability
SBPV: Systolic blood pressure variability
DBPV: Diastolic blood pressure variability
HRV: Heart rate variability
SBP: Systolic blood pressure
DBP: Diastolic blood pressure
HR: Heart rate
SDNN: Standard deviation of NN interval
CV: Coefficient variation
LF-nu: Low-frequency normalised unit
HF-nu: High-frequency normalised unit
TP: Total power
LF/HF: Ratio of LF and HF
PD: Panic disorder
MDD: Major depressive disorder
GAD: General anxiety disorder
ABPM: Ambulatory blood pressure monitoring
HBPM: Home blood pressure monitoring
LOD: Late-onset depression
EOD: Early onset depression
SD: Standard deviation
BP: Blood pressure
LLE-SBP: Largest Lyapunov exponent-systolic blood pressure
LLE-DBP: Largest Lyapunov exponent-diastolic blood pressure
LLE-HR: Largest Lyapunov exponent-heart rate
BPvi, Log_10_: ((BP detrended variance/mean BP^2^)/(HR detrended variance/mean HR^2^))
RRI: RR-interval
SAP: Systolic arterial pressure
DAP: Diastolic arterial pressure
